# *Bifidobacterium animalis* Supplementation Improves Intestinal Barrier Function and Alleviates Antibiotic-Associated Diarrhea in Mice

**DOI:** 10.3390/foods14101704

**Published:** 2025-05-11

**Authors:** Xiaoyu Du, Mingkun Liu, Jingyu Li, Yue Liu, Shaoyang Ge, Haina Gao, Ming Zhang

**Affiliations:** 1 School of Food and Health, Beijing Technology and Business University, Beijing 100048, China; 2 School of Food and Biological Engineering, Hefei University of Technology, Hefei 230000, China

**Keywords:** antibiotic-associated diarrhea, *Bifidobacterium animalis*, intestinal mucosal barrier, microbiota

## Abstract

Probiotics have gained increasing recognition for their potential to mitigate antibiotic-associated diarrhea (AAD). However, the precise mechanisms underlying their effects remain unclear. This study developed a mouse model of AAD using ceftriaxone to investigate the alleviating effects and mechanisms of *Bifidobacterium animalis* A6 (A6). The findings indicated that A6 supplementation effectively attenuated ceftriaxone-associated diarrhea in mice. The morphological damage to the villi and crypts was partially restored and more neatly reorganized following the A6 intervention. Additionally, intestinal morphology observations revealed a significant increase in the thickness of the mucus layer in the A6-treated group. Further examination of key regulatory genes associated with mucus secretion demonstrated that the A6 intervention effectively upregulated the expression of *mucin1*, thereby reinforcing the mucus layer. Concurrently, the A6 intervention upregulated the expression of the *AQP4* and *SLC26A3* genes in the intestine, which is responsible for restoring water absorption capacity in AAD mice. Additionally, the A6 treatment reduced ceftriaxone-induced harm to the intestinal microbiota of the mice, boosting beneficial bacteria like *Bacteroidales*, *Akkermansia*, *Bifidobacterium*, and *Lactobacillus*. Overall, this study offers valuable insights into the potential therapeutic role of A6 in restoring intestinal homeostasis and alleviating symptoms associated with AAD.

## 1. Introduction

Antibiotic-associated diarrhea (AAD) has emerged as a global concern due to the excessive use of antibiotics, particularly lincomycins and β-lactam antibiotics [1]. It is estimated that approximately 5% to 35% of patients experience AAD following antibiotic treatment [2]. Cephalosporins, a subclass of β-lactam antibiotics, are notably associated with the disruption of gut microbiota and the compromise of intestinal barrier function. Consequently, there is an urgent need to identify novel strategies to mitigate AAD, especially cases induced by cephalosporin antibiotics.

The alteration of gut microbiota by cephalosporins can lead to an imbalance in the microbial ecosystem, increasing the risk of infection by potential pathogens [3,4]. Therefore, research aimed at alleviating AAD has concentrated on strategies to regulate gut microbiota and reinforce the gut barrier. Various functional components in food products have been reported to have AAD-improving effects and have received widespread attention due to their safety for consumption and low price. Polysaccharides derived from natural foods have demonstrated potential in alleviating AAD [5]. Additionally, the application of glucose oxidase as an antibiotic alternative has been investigated, with findings suggesting its capacity to alleviate diarrhea and enhance intestinal health by augmenting antioxidant properties and preserving gut integrity [6].

In functional food ingredients, in addition to polysaccharides, probiotics have garnered increasing recognition for their potential to mitigate AAD through the modulation of intestinal microbiota, metabolites, and the mucinosal barrier. These live microorganisms confer health benefits to the host. Empirical evidence has demonstrated the efficacy of specific probiotic strains, including *Lactobacillus GG* and *Saccharomyces boulardii*, in the prevention and treatment of AAD [7]. One study underscores the role of probiotics in re-establishing the balance of gut microbiota disrupted by antibiotic therapy. Probiotics facilitate an increase in the abundance of beneficial bacteria while inhibiting the proliferation of pathogenic species, thereby reinstating the microbial equilibrium essential for gut health [8]. This modulation of gut microbiota is critical, as it aids in preserving the integrity of the intestinal barrier and prevents the translocation of harmful bacteria and toxins into the bloodstream [9].

Furthermore, probiotics have been shown to enhance the production of short-chain fatty acids (SCFAs), such as butyrate, which are crucial for maintaining intestinal barrier function and attenuating inflammation [10]. SCFAs serve as a crucial energy source for colonocytes and contribute to the reinforcement of tight junctions between epithelial cells, thereby enhancing the integrity of the mucinosal barrier [11]. Beyond their impact on the microbiota and metabolites, probiotics also exert a significant influence on the immune response. They have the capacity to modulate the expression of cytokines and other immune markers, which can lead to a reduction in inflammation and an improvement in the overall immune function of the gut [11]. Despite these promising findings, the precise molecular pathways through which probiotics exert their effects remain under investigation. Further research is required to fully elucidate the interactions between probiotics, the gut microbiota, and the host immune system to optimize their application in clinical settings [12].

*Bifidobacterium animalis* (*B. animalis*) is known for its ability to survive the harsh conditions of the gastrointestinal tract, including resistance to gastric acid and bile, which makes it a more effective probiotic for promoting gut health than other bifidobacteria [13]. This species was also noted for its ability to adhere to intestinal epithelial cells, which is crucial for colonization and exerting beneficial effects on the host [14]. *Bifidobacterium animalis* A6 (A6), isolated from the feces of the long-lived population in Bama, Guangxi, China, has demonstrated the ability to reduce inflammation and regulate intestinal SCFAs [15]. In addition, our previous research has demonstrated that A6 has the ability to relieve periodontal inflammation, bone loss, and obesity [16,17]. All of these results demonstrate that A6 has good potential for improving gut health. The primary objective of the present study was to explore the potential effects and underlying mechanisms of A6 in AAD. A ceftriaxone-induced AAD model was employed for this investigation. Understanding these mechanisms will not only improve the therapeutic application of probiotics for AAD but also potentially extend their use to other gastrointestinal disorders.

## 2. Materials and Methods

### 2.1. Mouse Model of AAD

**Figure 1:**
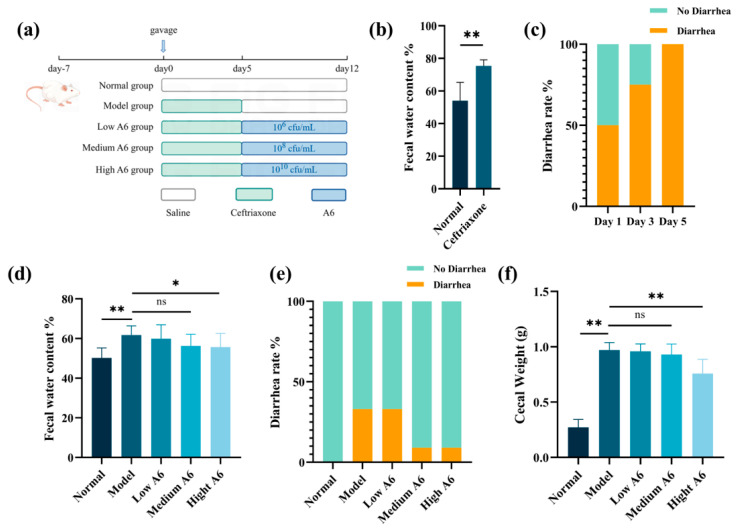
Effects of A6 on diarrhea indexes in AAD mice: (**a**) study design; (**b**) changes in fecal water content in mice between the normal group (*n* = 12) and the ceftriaxone-treated group (*n* = 48); (**c**) changes in the diarrhea ratio during ceftriaxone treatment; (**d**) changes in the fecal water content of mice in different treatment groups on day 12 (*n* = 12); (**e**) changes in the diarrhea ratio of mice in different treatment groups on day 12 (*n* = 12); (**f**) weight of the mouse cecum (*n* = 12). The error bar represents the mean ± SE. Means marked with asterisks are significantly different (* *p* < 0.05; ** *p* < 0.01; ns *p* > 0.05).

A cohort of 60 six-week-old male BALB/c mice (body weight 18–22 g) was procured from Vital River Laboratory Animal Technology Co., Ltd. (Beijing, China). All the mice were housed in an animal holding room under controlled conditions of 21 ± 2 °C, 50 ± 10% humidity, and a 12 h light/dark cycle. All the mice were given the basal diet and water ad libitum. Following a one-week acclimatization period, the mice were randomly assigned into five distinct groups, as depicted in Figure 1a, including the normal group (*n* = 12), the AAD model group (model, *n* = 12), and three A6 treatment groups differentiated by dosage: low dose (2 × 10^5^ cfu/day, low A6, *n* = 12), medium dose (2 × 10^7^ cfu/day, medium A6, *n* = 12), and high dose (2 × 10^9^ cfu/day, high A6, *n* = 12). All groups, except for the normal group, received a daily administration of 0.2 mL of 250 mg/mL ceftriaxone for five consecutive days to induce the diarrhea model. The criteria for successful modeling were that all the mice in the model group had diarrhea and that the ratio of dry to wet weight of feces was statistically different compared with the normal group. Upon successful establishment of the model, the three A6 intervention groups were administered a daily gavage of 0.2 mL A6 suspension at concentrations of 1 × 10^6^ cfu/mL, 1 × 10^8^ cfu/mL, and 1 × 10^10^ cfu/mL, respectively, over a period of seven days, while the model group received 0.2 mL of sterile saline. Throughout the experimental duration, daily measurements of body weight, food intake, and observations of diarrhea symptoms were meticulously recorded. The experimenters were blinded when assessing outcomes. The animal use license was SCXK 2012-0001, and the experimental protocol was approved by the Experimental Animal Committee of China Agricultural University (No. AUJ21903204).

The fecal water content was detected on days 1, 3, 5, and 12 of the experimental period. Individual mice were placed in clean cages and fasted for 1 h. All fresh fecal pellets excreted during this 1 h period were collected into clean centrifuge tubes. The initial dry weight was measured immediately. The samples were subsequently dried at 105 °C for 2 h until the weight change was below 1%, and the dry weight was then recorded. The fecal water content was calculated accordingly: Fecal water content (%) = (Fecal weight before drying − Fecal weight after drying)/Fecal weight before drying × 100.

The diarrhea rate was detected on days 5 and 12, and the diarrhea rate was measured. The calculation formula is as follows: Diarrhea rate (%) = (The number of mice with diarrhea in the group)/(The total number of mice in the group) × 100.

### 2.2. Histological Analysis of Colon Tissue

At the conclusion of this study, the mice were euthanized and dissected. In accordance with the methodology outlined by Wu, Y et al. [18], the cecum was excised and weighed, and segments measuring 2 cm from both the ileum and proximal colon were collected, immersed, and fixed in 4% paraformaldehyde and carnot fixative for hematoxylin/eosin (HE) staining and Alcian Blue–Periodie Acid Schiff (AB-PAS) staining, respectively. The cecum was weighed, and 2 cm segments from the ileum and proximal colon were collected and fixed in 4% paraformaldehyde (for H&E staining) or Carnoy’s fixative (for AB-PAS staining). Tissue sections (5 μm) were processed through parallel H&E and AB-PAS staining protocols, including baking (65 °C), deparaffinization, rehydration, and staining (H&E: Mayer’s hematoxylin/eosin; AB-PAS: Alcian Blue [pH 2.5]/periodic acid/Schiff’s reagent), followed by dehydration and mounting. The intestinal tissues of 6 mice were randomly selected for histological examination, and the remaining intestinal tissue was used for subsequent RT-qPCR analysis.

### 2.3. Analysis Using 16S rRNA Gene Sequencing

Sequencing was carried out by Shanghai Meiji Biomedical Technology Co., Ltd. (Shanghai, Beijing). Microbial DNA from mouse fecal samples was extracted using the PF Mag-Bind Stool DNA Kit. The V3-V4 regions of 16S rRNA genes were amplified using primers 338F (5′-ACTCCTACGGGAGGCAGCAG-3′) and 806R (5′-GGACTACHVGGGTWTCTAAT-3′). The PCR conditions were as follows: 95 °C 3 min; 27 cycles of 95 °C 30 s, 55 °C 30 s, and 72 °C 45 s; and final extension 72 °C 10 min. PCR products were purified by agarose gel electrophoresis and sequenced on the Illumina MiSeq PE300 platform. Raw data were quality-filtered with fastp, assembled by FLASH, and clustered into ASVs (97% similarity) using UPARSE. Taxonomic annotations were performed using the Silva 16S rRNA database (v138).

### 2.4. Determination of Fecal SCFAs

Fecal samples (25 mg) were homogenized with 500 μL of 0.5% phosphoric acid solution by cryogenic grinding (50 Hz, 3 min × 2 cycles), followed by ultrasonication (10 min) and centrifugation (13,000× *g*, 15 min, 4 °C). The supernatants were mixed with 0.2 mL n-butanol containing internal standard (2-ethylbutyric acid, 10 μg/mL), vortexed (10 s), ultrasonicated (10 min), and centrifuged again (13,000× *g*, 5 min, 4 °C). The supernatants were analyzed by headspace GC-MS (Agilent 7200 GC-QTOF, HP-FFAP column; helium 1.0 mL/min; injector 260 °C; split 10:1; 1 μL injection; oven 50–240 °C; EI 70 eV, SIM mode).

### 2.5. RT-qPCR for Intestinal Gene Expression

RT-qPCR was performed to detect the gene expression of mucin-forming and water–electrolyte channels. Intestinal tissue cDNA, obtained through extraction and reverse transcription, was subjected to quantitative PCR to measure the gene expression levels of mucin-forming (*mucin*1, *mucin*4) and water–electrolyte channels (*AQP4*, *NHE3*, and *SLC26A3*). The primer sequences for these genes are provided in Table 1.

RNA was extracted from colon tissues stored at −80 °C using TRIzol, purified with chloroform/isopropanol, and dissolved in DEPC-treated water. RNA quality (A260/A280 > 1.8) was assessed using a NanoDrop 2000 (Thermo, Waltham, MA, USA). Reverse transcription was performed with HiScript IV All-in-One Ultra RT SuperMix(Abcam, Shanghai, Beijing) (5 μL RNA/reaction; 37 °C, 15 min; 60 °C, 10 min; 95 °C, 3 min). Quantitative PCR conditions were performed based on the manufacturer’s protocol using the TaKaRa Real-Time PCR Kit (Takara, Shiga, Japan). SYBR Green functioned as the fluorescent probe. β-actin served as the reference gene to rectify the expression of target genes. The 2^−ΔΔCt^ method was used to calculate the relative gene expression levels. All reactions were performed in triplicate.

### 2.6. Statistical Analysis of Data

The experimental data were analyzed using GraphPad Prism (version 8.0.1). The results are presented as mean ± standard deviation (SD). One-way ANOVA and Dunnett’s multiple comparison tests served as tools for comparing multiple groups. A *p*-value of less than 0.05 was considered statistically significant, and a *p*-value of less than 0.01 was considered statistically highly significant.

## 3. Results

### 3.1. A6 Supplementation Improved Diarrhea Status in AAD Mice

To assess the effects of A6 on mitigating antibiotic-associated diarrhea, we utilized the fecal water content and the percentage of diarrhea in the mice as indicators of diarrhea status. The fecal water content serves as a marker for diarrhea in mice, typically increasing when diarrhea is present. In comparison to the normal group, the ceftriaxone treatment significantly elevated the fecal water content from 54.1% to 75.3% (*p* < 0.01) (Figure 1b). However, the A6 intervention dose-dependently reversed these effects. Compared with the model group, high doses reduced the fecal water content by 6.82% (*p* < 0.01) (Figure 1d). Furthermore, the ceftriaxone treatment induced diarrhea in a time-dependent manner, with the diarrhea rate reaching 100% by day 5 (*p* < 0.01) (Figure 1c). The high-dose A6 achieved significant clinical improvement in diarrhea rates. The comparative analysis revealed that 33% of the mice in the model group exhibited diarrhea symptoms, whereas only 8% of the mice in the high-dose A6 group showed diarrhea. The 25% reduction in the diarrhea rate demonstrated a significant protective effect of A6 supplementation (Figure 1e). Moreover, high-dose A6 significantly reversed the caecum swelling effect in the ceftriaxone-induced mice (Figure 1f) (*p* < 0.01). Overall, these findings indicated that A6 effectively attenuated ceftriaxone-associated diarrhea in the mice.

### 3.2. A6 Intervention Enhanced the Intestinal Mucus Barrier in AAD Mice

**Figure 2:**
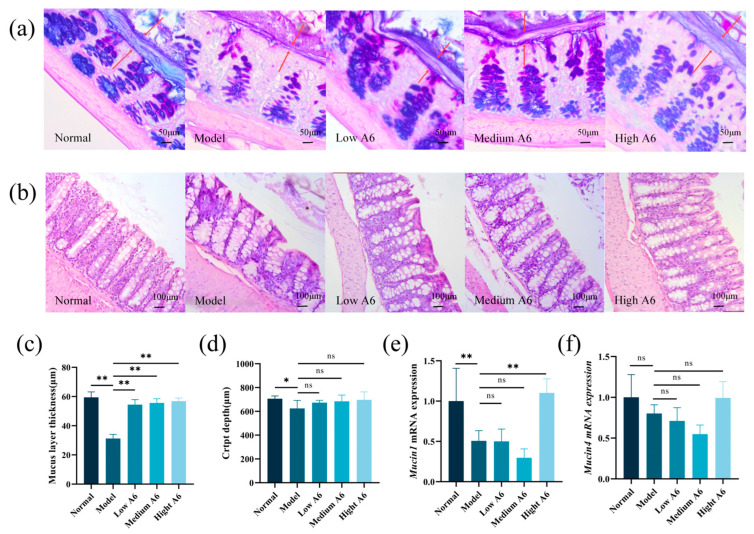
*A6* enhanced the mucus barrier and the expression of mucin-forming genes in AAD mice: (**a**) AB-PAS staining of proximal colon tissues (magnification: 40 × 5; red arrows: colonic mucus layers); (**b**) HE staining of colon tissues of mice in different treatment groups in the recovery period (magnification: 20 × 5); (**c**) effect of A6 intervention on the thickness of the mucinous layer in the proximal colon of mice (*n* = 6); (**d**) effect of A6 intervention on the depth of crypts in the colon of mice (*n* = 6); (**e**,**f**) *Mucin1* and *mucin4* gene expression in mice in different treatment groups (*n* = 6). The error bar represents the mean ± SE. Means marked with asterisks are significantly different (* *p* < 0.05; ** *p* < 0.01; ns *p* > 0.05).

The mucus layer serves as the primary protective barrier of the mucosa overlying the intestinal epithelial cells, and its integrity is crucial for maintaining health. To examine the impact of A6 on mucus thickness, HE staining was conducted on colonic tissue samples. As illustrated in Figure 2a, the colonic mucus layers in the normal group were intact. Conversely, the mucus layer in the model group was thinner. Notably, the A6 treatment resulted in a significant increase in mucus layer thickness in the AAD mice (*p* < 0.01) (Figure 2c). These findings suggested that supplementation with A6 enhances the structural solidity of the colonic mucus layer in AAD mice.

Figure 2b demonstrates that the colonic tissues in the normal group exhibited intact and neatly arranged crypts with abundant goblet cells. In contrast, the ceftriaxone treatment disrupted the uniform arrangement and integrity of the colon villi, whereas A6 administration ameliorated the integrity of the colon villi. Interestingly, the crypt depth in the model group was reduced compared to the normal group, while A6 administration increased the crypt depth in the colon, although there was no significant difference (Figure 2d).

### 3.3. A6 Intervention Modulated the Mucin-Forming Gene in AAD Mice

The gel-forming mucin constitutes a crucial structural and functional element of the mucus barrier. This study further explored the effects of A6 supplementation on the colon mucus barrier in AAD mice, employing RT-qPCR analysis to assess the expression levels of mucin-forming genes. As illustrated in Figure 2e,f, the ceftriaxone treatment resulted in a reduction in *mucin1* and *mucin4* mRNA expression compared to the normal group. Notably, compared with the model group, high-dose A6 supplementation significantly upregulated mucin1 mRNA expression (2.17-fold increase, *p* < 0.01) relative to the model group, while mucin4 exhibited an increasing tendency (1.24-fold increase, *p* = 0.2352). These results suggest that A6 may exert its effects through selective activation of the MUC1 pathway.

### 3.4. A6 Supplementation Restored Gut Microbial Dysbiosis in AAD Mice

**Figure 3:**
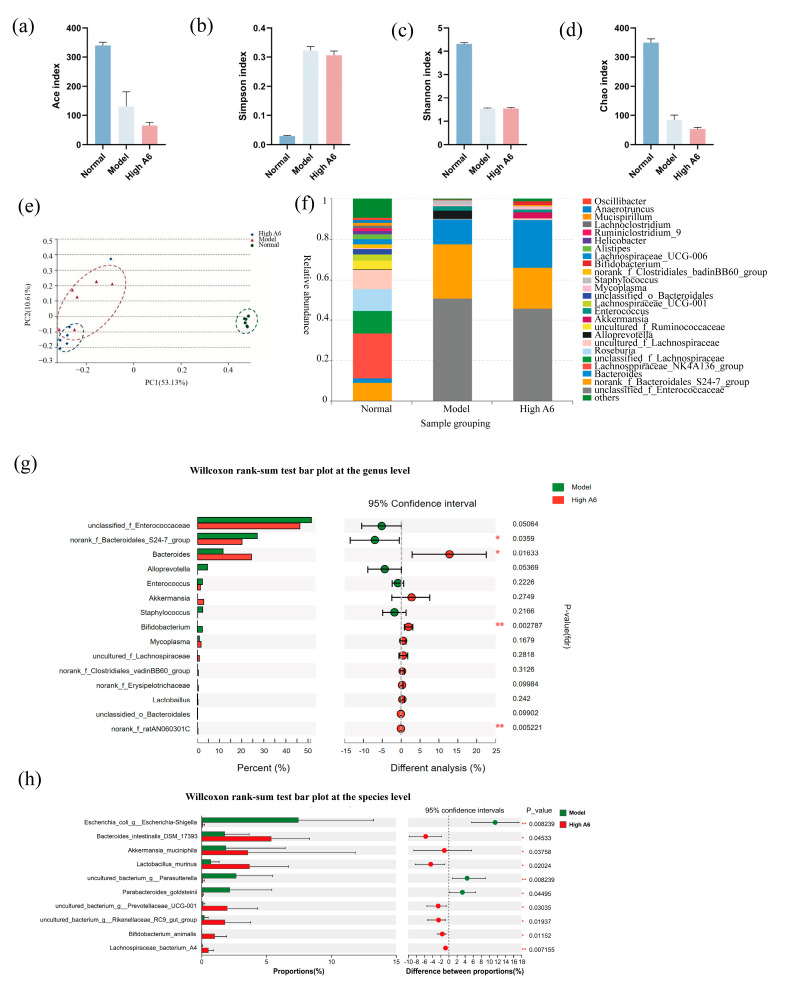
Effects of *A6* on the intestinal microbiota of AAD mice: (**a**–**d**) alpha diversity analysis; (**e**) UniFrac PCoA analysis of the intestinal microbiota of mice in different treatment groups; (**f**) species relative abundance at the genus level in different groups; (**g**,**h**) differential microorganisms at the genus and species levels between the model group and the high-dose A6 group. The error bars represent the mean ± SE, n = 6. Means marked with asterisks are significantly different (* *p* < 0.05; ** *p* < 0.01).

To elucidate the influence of A6 on gut microbial dysbiosis, 16S rRNA sequencing was utilized. Alpha diversity indices, including Ace, Chao, Shannon, and Simpson, serve as indicators of species richness and diversity within microbial communities. In this study, the Ace, Chao, and Shannon indices were significantly reduced, while the Simpson index was significantly elevated in the model group compared to the control group. No significant differences were observed in the A6 treatment group relative to the model group. The β-diversity of the gut microbiota was assessed through Principal Coordinates Analysis (PCoA) using the Bray–Curtis distance metric. Our findings demonstrated significant variations in distance distributions across the different groups. The PCoA visualization revealed that PC1 (53.13%) and PC2 (10.61%) represent the principal and secondary variances in the dataset, respectively. Both PC1 and PC2 demonstrate substantial differences between the model and high A6 groups. The analysis of PC1 clearly indicates significant differences between the normal/model groups and the high A6 group, whereas PC2 distinctly demonstrates notable differences between the model group and the high A6 group (Figure 3e). The predominant gut microbiota in the model group was *Enterococcaceae*, whereas in the A6 group, it was *Bacteroidales*, *Akkermansia*, *Bifidobacterium*, and lactobacilli (Figure 3f). Subsequently, we examined the differential microorganisms at the genus and species levels between the model group and the high-dose A6 group. At the genus level, the high dose of the A6 intervention significantly elevated the abundance of *Bacteroides* and *Bifidobacterium*, while other beneficial bacteria, such as *Akkermansia* and *Lactobaillus*, showed an elevated trend (Figure 3g). At the species level, the intervention of A6 significantly elevated the abundance of *Bacteroides intestinalis*, *Akkermansia maciniphila*, *Lactobacillus murinus*, and *B. animalis* (Figure 3h). These results proved that *A6* supplementation renovated ceftriaxone-induced gut microbiota disturbances and elevated the levels of various beneficial bacteria in the mouse intestine.

### 3.5. A6 Supplementation Upregulated SCFAs in AAD Mice

**Figure 4:**
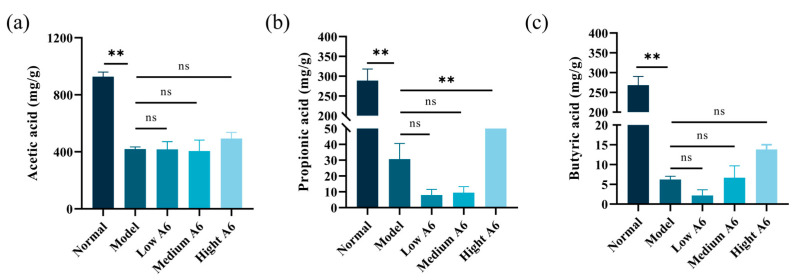
Level of SCFAs in the feces of each group: (**a**) acetic acid content (mg/g); (**b**) propionic acid content (mg/g); (**c**) butyric acid content (mg/g). The error bars represent the mean ± SE, n = 6. Means marked with asterisks are significantly different (** *p* < 0.01; ns *p* > 0.05).

SCFAs, as metabolites produced by gut microorganisms, function as crucial signaling molecules between these microorganisms and their hosts. In comparison to the normal group, the ceftriaxone treatment resulted in a significant reduction in the levels of acetic acid, propionic acid, and butyric acid. Conversely, the high-dose A6 group demonstrated increased levels of these acids compared to the model group (Figure 4). It is noteworthy that the concentrations of propionic acid (*p* < 0.01) were significantly elevated in the model group (Figure 4b). Based on the analysis of intestinal microbiota, we proposed that the observed increase in propionic acid may be attributed, in part, to a heightened abundance of *Bifidobacteria* and *Bacteroidales*. This increase likely enhanced the microbial capacity for carbohydrate metabolism, thereby leading to elevated production of SCFAs. These findings suggest that A6 has the potential to counteract the decline in propionic acid content in the feces of AAD mice. Nevertheless, the upregulation of A6 for propionic acid was far from normal, and there was no significant relief from the decline in acetic and butyric acids.

### 3.6. A6 Supplementation Recovered Water and Electrolyte Transport in AAD Mice

**Figure 5:**
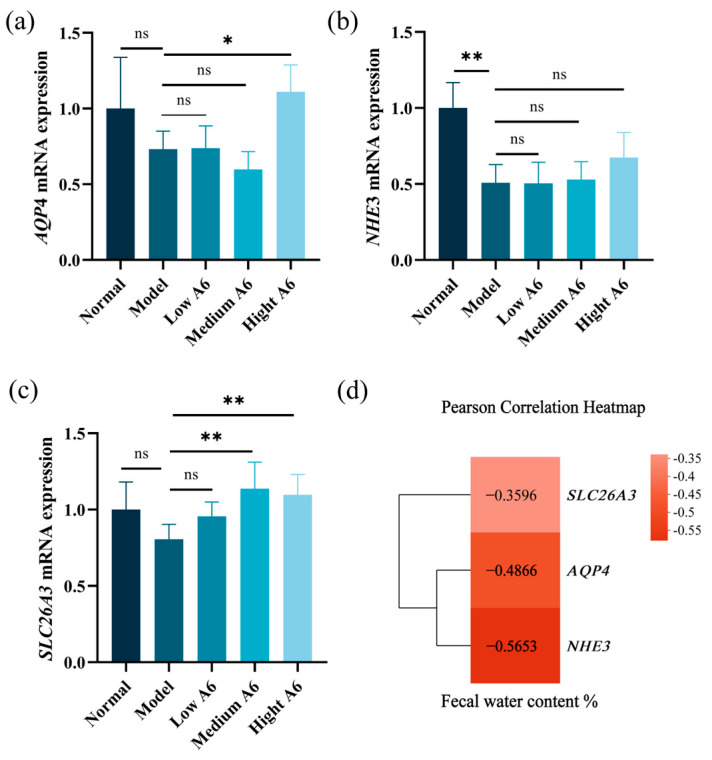
Water and electrolyte transport gene expression of mice in different treatment groups. (**a**–**c**) The mRNA expression of *AQP4*, *NHE3*, and *SLC26A3*; (**d**) Pearson correlation heatmap between gene expression levels and fecal water content. The error bars represent the mean ± SE, *n* = 6. Means marked with asterisks are significantly different (* *p* < 0.05; ** *p* < 0.01; ns *p* > 0.05).

Several water and electrolyte transporters have been implicated in the pathogenesis of diarrhea [24]. To assess the impact of A6 on water and electrolyte transport, we measured the expression levels of *AQP4*, *NHE3*, and *SLC26A3* using RT-PCR (Figure 5). *AQP4*, which is primarily expressed on the brush border of colonic epithelial cells, plays a key role in water reabsorption in the intestine. Compared to the normal group, the mRNA expression level of *AQP4* was reduced in the model group (Figure 5a). However, supplementation with A6 led to an upregulation of *AQP4* gene expression, especially with high-dose A6 supplementation (*p* < 0.05). *SLC26A3* works synergistically with *NHE3* to promote Na^+^ absorption in the lower gastrointestinal tract and enhance passive water absorption. As shown in Figure 5b, the gene expression levels of *NHE3* in the model group were significantly reduced (*p* < 0.01), and the expression of the two genes showed a certain upward trend after the A6 intervention, but the difference was not significant (*p* > 0.05). In addition, the expression level of the *SLC26A3* gene was significantly increased by the medium- and high-dose A6 interventions (*p* < 0.01). These findings suggested that supplementation with A6 may enhance the water reabsorption capacity of epithelial cells in the intestinal lumen.

## 4. Discussion

This study created a mouse model of AAD using ceftriaxone to explore the effects of probiotic A6. A6 supplementation significantly reduced diarrhea and partially restored villi and crypt morphology. It increased mucus layer thickness by upregulating *mucin1* genes. In addition, the A6 intervention upregulated the expression of the *AQP4* and *SLC26A3* genes in the intestine, which is responsible for restoring water absorption capacity in AAD mice. A6 also improved intestinal microbiota, boosting beneficial bacteria and increasing propionic acid levels.

Antibiotic treatment induces intestinal dysbiosis, featuring prominent overgrowth of drug-resistant opportunistic pathogens (particularly *Enterococcus* spp., *Clostridium difficile*, *Clostridium perfringens*, and *Staphylococcus aureus*) [25]. This microbial imbalance exerts multifaceted detrimental effects: depletion of beneficial SCFA-producing commensal bacteria, suppression of intestinal tight junction proteins (occludin and ZO-1), and hyperactivation of the pro-inflammatory TLR4/NF-κB signaling pathways. Collectively, these pathological alterations disrupt microbial homeostasis and severely compromise intestinal barrier integrity, ultimately leading to the development of AAD [26]. Ceftriaxone sodium, a third-generation cephalosporin antibiotic, exhibits broad-spectrum antimicrobial activity. It demonstrates high efficacy towards anaerobic bacteria; however, *Bacteroides fragilis* and *Enterococcus* species remain resistant to it [27,28,29].

Several probiotic strains, including *Lactobacillus rhamnosus* GG, *Saccharomyces boulardii*, and *Bifidobacterium lactis*, have been clinically validated for the prevention or treatment of AAD. *Lactobacillus rhamnosus* GG (LGG) demonstrates significant therapeutic efficacy in ameliorating intestinal dysfunction induced by combined metronidazole–neomycin–vancomycin administration. The probiotic strain specifically upregulates the expression of key ion transport proteins, including *NHE3*, *SLC26A3*, and *AQP4*, thereby restoring luminal water–electrolyte homeostasis in the antibiotic-treated gut [23]. Furthermore, the multi-strain probiotic formulation VSL#3 constituent bacterial strains secrete antimicrobial peptides (e.g., bacteriocins) that directly inhibit colonization of *Clostridium difficile* and significantly suppress Toll-like receptor (TLR) signaling pathway activation (*p* < 0.01), resulting in marked downregulation of pro-inflammatory cytokines (IL-8 and TNF-α). These combined actions lead to a dramatic reduction in AAD incidence [30]. Furthermore, research by Chai Jianhua et al. [31] revealed that adjunctive therapy with a quadruple viable *Bifidobacterium* preparation significantly facilitated recovery from AAD in infants and young children, with a commendable safety profile. *Bifidobacterium* is recognized as a crucial probiotic within the gut microbiota, known for its ability to enhance intestinal microflora, bolster immune function, and address intestinal disorders, such as diarrhea and enteritis.

Notably, our study reveals that a high dose of A6 demonstrates remarkable advantages in both mucosal repair and microbial ecological remodeling. Histopathological analysis demonstrates that A6 administration effectively restores colonic mucus layer thickness. Furthermore, metagenomic sequencing analysis indicates that A6 significantly enhances the colonization and expansion of mucin-degrading bacteria (e.g., *Akkermansia muciniphila*) and lamina propria commensals (e.g., *Lactobacillus murinus*). These findings suggest that A6 reconstructs intestinal barrier function through ecological-level modulation of symbiotic microbiota interactions, resulting in significant attenuation of AAD manifestation.

The mucinous layer is an essential component of intestinal barrier integrity, functioning as the primary defense mechanism against gut bacteria [32]. It safeguards epithelial cells from potentially harmful antigens and molecules while also serving as a lubricant for intestinal peristalsis [33]. Damage or thinning of the mucinous layer compromises its physical barrier function, thereby facilitating the access of pathogens and harmful substances to epithelial cells, which can result in intestinal dysfunction and AAD [34]. A compromised intestinal barrier allows for pathogen invasion, which is a principal cause of diarrhea in AAD models [8]. Additionally, disruption of the mucinous layer can lead to dysbiosis of the gut microbiota, further aggravating intestinal barrier dysfunction and perpetuating a detrimental cycle [35]. Consequently, probiotics that enhance the thickness of the mucinous layer and restore intestinal barrier function may offer therapeutic benefits in alleviating AAD. Previous research has demonstrated that treatment with *Bacillus fragilis* can restore epithelial tissue organization and enhance barrier function, thereby ameliorating gastrointestinal symptoms in AAD rat models [8]. Additionally, dietary hesperetin has been shown to effectively reorganize goblet cells and increase the thickness of the mucinous layer, thereby reducing the likelihood of bacterial contact with the epithelium [36]. In our study, we observed a significant reduction in the colonic mucinous layer of the model group mice, with its thickness being only half that of the normal group. Following supplementation with A6, the colonic mucinous layer exhibited a more stable structure, with its thickness increasing in a dose-dependent manner, significantly surpassing that of the model group. These findings suggest that A6 plays a protective role in maintaining the integrity of the mucinous layer and intestinal barrier function.

The thickness of the mucinous layer is closely associated with goblet cells in the intestine, as these cells secrete large amounts of mucins, which form the structural framework of the mucinous layer [37]. Mucins are a class of high-molecular-weight glycoproteins that are either distributed on cell membranes or secreted into the intestinal lumen [38]. To date, 15 types of mucins have been identified, with mucin1 being the most prominent. Notably, *mucin1* is highly expressed on the apical surface of most polarized epithelial cells [39]. Beyond its roles in lubricating and protecting intestinal epithelial tissues, mucin1 also facilitates cell adhesion and signal transduction [40]. Mucin4, a heterodimeric membrane mucin [41], comprises two tightly linked subunits and is the predominant mucin expressed in normal human intestinal epithelium [42]. Previous studies have demonstrated that *Bifidobacterium* can influence the intestinal mucinous barrier. Our experimental results demonstrated that high-dose *A6* upregulated the expression of mucin1 (2.17-fold increase, *p* < 0.01) in the proximal colon of AAD mice, increased mucinous layer thickness, strengthened the mucinous barrier, and alleviated intestinal mucinosal damage. These findings underscore the potential of A6 as a therapeutic agent for mucin barrier restoration.

SCFAs play a crucial role in promoting the maturation of intestinal barrier function in healthy individuals and alleviating intestinal barrier damage in diseased hosts [43]. A reduction in luminal SCFAs is one of the pathogenic mechanisms underlying AAD [44]. Compared to the normal group, the model group exhibited a sharp decrease in acetic acid, propionic acid, and butyric acid. Compared to the model group, the high-dose A6 group exhibited elevated levels of propionic acid. Propionic acid and butyric acid, the key SCFAs, are primarily produced in the colon through the microbial fermentation of undigested dietary fibers and carbohydrates [45]. Consistent with our findings, Liu et al. demonstrated that elevated propionate and butyrate concentrations significantly enriched SCFA-producing genera, particularly Bifidobacterium, via enhanced cross-feeding interactions in the colonic niche [46]. One study highlighted the role of *Bifidobacteria animalis* in modulating the gut microbiota and enhancing SCFA production through the fermentation of arabinoxylan-based substrates [47]. It has been shown that members of *Bifidobacteria* can alter the metabolic activity of SCFA-producing bacteria by cross-feeding [48]. Additionally, *Bifidobacteria animalis* has been shown to interact with other gut bacteria, such as *Bacteroides* species, in co-fermentation processes [49]. *Bacteroides*, one of the major producers of propionic acid in the gut, not only efficiently utilizes complex polysaccharides but also optimizes propionic acid production through synergistic interactions with other gut microbes. Therefore, we proposed that the observed increase in propionic acid and butyric acid may be attributed, in part, to a heightened abundance of *Bifidobacteria* and *Bacteroides*. Propionic acid and butyric acid serve as a carbon and energy source for colonic epithelial cells [50]. They influence the proliferation, differentiation, and function of intestinal mucosal cells, thereby regulating mucus layer thickness and improving intestinal barrier function [51]. These findings highlight the importance of propionic acid in maintaining gut health and its potential therapeutic role in mitigating AAD-related intestinal barrier damage.

SCFAs are integral to the regulation of water and electrolyte transport in intestinal epithelial cells [52,53]. Specifically, propionic acid enhances the metabolic activity of colonic epithelial cells by serving as an energy source and facilitating *AQP4*-mediated water transport [54]. Concurrently, these acids influence the expression and function of *NHE3* and *SLC26A3*, either through the activation of G protein-coupled receptors or by acting directly as energy substrates [55]. This process contributes to the absorption of Na^+^ and Cl^−^, thereby creating an osmotic pressure gradient that drives the passive absorption of water [56,57]. In our study, the administration of a high dose of A6 significantly upregulated *AQP4* expression compared to the model group. These findings suggested that A6 may mitigate diarrhea by enhancing the capacity of epithelial cells to reabsorb water from the intestinal lumen, consequently reducing the fecal water content. This mechanism underscores the potential of A6 as a therapeutic agent for improving intestinal water and electrolyte balance.

AAD is typically accompanied by significant dysbiosis of the gut microbiota, characterized by a reduction in beneficial bacteria, the proliferation of opportunistic pathogens, and disruption of the mucosal barrier. In this study, high-dose A6 supplementation resulted in a notable decrease in the abundance of opportunistic pathogens, such as *Enterococcus*, *Escherichia spp.*, *Parasutterella*, and *Parabacteroides goldsteinii*, which were prevalent in the model group. The suppression of these taxa is significant, as their overrepresentation is often linked to dysbiosis and gastrointestinal disorders [58]. And the overgrowth of these pathogens can impair the intestinal mucus layer, reduce colonization resistance, and promote bacterial adhesion, tissue invasion, inflammation, and diarrhea. Antoni PA et al. [59] demonstrated that treatment with low-dose cephalosporins combined with *Enterococcus faecium* gavage reduced *Mucin2* secretion and nearly eliminated the mucinous layer in mice. This finding aligns with the observed reduction in the mucinous layer in the AAD mice in our study, further confirming that microbiota disruption affects mucin expression and the defensive function of the mucinous barrier. Notably, when *Escherichia coli* coexists with *Enterococcus faecalis*, the expression of virulence genes (*espA*, *espB*) in *E. coli* is significantly upregulated. This intermicrobial synergy amplifies virulence phenotypes through mechanisms such as the disruption of intestinal barrier integrity and the induction of epithelial apoptosis, ultimately triggering diarrhea [60]. *Parasutterella* leads to a significant reduction in fecal deoxycholic acid (DCA) and lithocholic acid (LCA) levels, potentially aggravating inflammation [61]. *P. goldsteinii*, initially isolated from the feces and abdominal tissues of patients with appendicitis, peritonitis, and intra-abdominal abscesses, is hypothesized to act as an opportunistic pathogen under certain conditions, contributing to inflammation or barrier dysfunction [62].

Dysbiosis of the intestinal microbiota is a characteristic feature of AAD, and restoring gut microbiota homeostasis is essential for effective AAD treatment. Lucy McDonnell et al. comprehensively analyzed the strains that typically change in antibiotic-induced microbiota dysbiosis, with decreases in *Bifidobacteria* and lactobacilli and increases in *Clostridium* abundance being the most frequently observed [63]. Parker also reported that a significant reduction in Proteobacteria (mainly the species *Akkermansia mucinophilia*) was frequently observed, which were particularly susceptible to antibiotics [64]. Similarly, research involving horses revealed that antibiotic administration led to a significant reduction in cellulolytic bacteria and lactobacilli, while allowing the proliferation of pathogens like *Salmonella* and *Clostridium difficile* [65]. In this study, ceftriaxone sodium treatment disrupted the gut microbiota in mice. The abundance of anaerobic bacteria, such as *Bifidobacterium, Akkermansia,* and *Lactobaillus*, drastically decreased and nearly disappeared. High-dose A6 intervention reversed these phenomena to some extent, which significantly elevated the abundance of *Bacteroides intestinalis, Akkermansia maciniphila, Lactobacillus murinus*, *B. animalis*, *Prevotellaceae_*UGG001, *Rikenellaceae_*RC9*_gut_group*, and *Lachnospiraceae _bacterim_*A4 (Figure 3h). We believe that changes in these key strains will have many positive effects on AAD. For example, *Akkermansia mucinophilia* has previously been recognized as having anti-inflammatory properties and improving intestinal barrier function [66]. It enhances intestinal barrier integrity by upregulating tight junction proteins (e.g., ZO-1 and occludin), stimulates mucus production through MUC2 promotion and MUC1 degradation, and exhibits anti-inflammatory properties [67]. Lactobacilli and *Bifidobacterium* were found to stimulate Cl^−^/HCO_3_^−^ and Na^+^/H^+^ exchange activities, which were crucial for water movement and absorption in the colon [68,69]. Notably, Lactobacilli are also recognized for their immunomodulatory effects, including the regulation of T-lymphocyte activity and macrophage polarization, which contribute to alleviating intestinal mucosal injury and regulating microbial homeostasis [70]. Moreover, *Bacteroides* spp. may provide some level of protection from invasive pathogens, such as a gut commensal [71]. Families like *Prevotellaceae* and *Rikenellaceae* have also been reported to have protective effects on intestinal health. One study indicated that *Prevotellaceae_*UCG-003 can promote the production of SCFAs (acetate, isobutyrate, and butyrate) to alleviate diarrhea [72]. The *Rikenellaceae* family has been associated with anti-inflammatory effects and the maintenance of gut barrier function. An increased abundance of *Rikenellaceae* correlates with reduced intestinal permeability and inflammation [73]. These results will provide the necessary evidence for the application of A6 in human clinical trials, especially in the regulation of microbiota dysbiosis.

However, based on the current results, we have not been able to accurately clarify how A6 interacts with specific gut bacteria and increases the levels of several of these beneficial bacteria. We hypothesized that A6 may promote the colonization and proliferation of other beneficial bacteria by cross-feeding through its specific carbohydrate metabolizing ability. *Bifidobacteria* have been estimated to use approximately 14.64% of the carbohydrate-active enzymes gene [74], which rely on their enzymatic breakdown and oligosaccharide transport systems, facilitating cross-feeding relationships with other bacteria, such as *Bacteroides* and lactobacilli, and other SCFA producers [75].

## 5. Conclusions

In summary, this study developed a mouse model of AAD using ceftriaxone to investigate the alleviating effects and mechanisms of A6. The intestinal epithelial tissue served as the primary focus of this research. We examined the fine structural morphology of villi and crypts, the absorption function of water and electrolyte ions, and the key defensive barrier of the mucus layer. These analyses were conducted to explore the specific mechanisms by which A6 mitigates AAD. The findings provide valuable insights into the potential therapeutic role of A6 in restoring intestinal homeostasis and alleviating AAD-related symptoms. And more importantly, this study provides potential strains for the prevention of AAD through functional food intake.

## Figures and Tables

**Table 1:** Oligonucleotide probes used in this study.

Genetics	Primer Sequence	Bibliography
β-actin	F: GGCTGTATTCCCCTCCATCG R: CCAGTTGGTAACAATGCCATGT	(Tang J, 2011) [19]
Mucin1	F: CTGTTCACCACCACCATGAC R: CTTGGAAGGGCAAGAAAACC	(Krik S, 2010) [20]
Mucin4	F: CAGCAGCCAGTGGGGGACAG R: CTCAGACACAGCCAGGGAACTC	(Hoebler E, 2006) [21]
AQP4	F: AGCATCGCTAAGTCCGTCTTC R: TCCTCCACCTCCATGTAGCTC	(Hardin JA, 2004) [22]
NHE3	F: TGGCCGGGCTTTCGACCACA R: GGGACCCACGGCGCTCTCCCT	(Gail C, 2013) [23]
SLC26A3	F: CACAAATTCAGAAGACGAACA R: GCATCAGCATTCCCTTTAAGTT	(Gail C, 2013) [23]

## Data Availability

The original contributions presented in this study are included in this article. Further inquiries can be directed to the corresponding author.
